# Parental Time of Returning Home From Work and Child Mental Health Among First-Year Primary School Students in Japan: Result From A-CHILD Study

**DOI:** 10.3389/fped.2018.00179

**Published:** 2018-07-02

**Authors:** Masashi Kizuki, Manami Ochi, Aya Isumi, Tsuguhiko Kato, Takeo Fujiwara

**Affiliations:** ^1^Department of Global Health Promotion, Tokyo Medical and Dental University, Tokyo, Japan; ^2^Japan Support Center for Suicide Countermeasures, Tokyo, Japan; ^3^Department of Social Medicine, National Center for Child Health and Development, Tokyo, Japan

**Keywords:** Adachi child health impact of living difficulty study, strengths and difficulties questionnaire, time of returning home from work, parenting, mediation analysis, Japan

## Abstract

**Introduction:** Child mental health is known to be influenced by parental work hours. Although literature suggests that parent-child interaction mediates the association, few studies have directly measured the parental time of returning home from work. We analyzed data from a school-based survey to examine the association between parental time of returning home from work and child mental health.

**Methods:** We used a sample of 2,987 first-year primary school students derived from the Adachi Child Health Impact of Living Difficulty (A-CHILD) study that examined the impact of family environment and lifestyle on child health in Adachi City, Tokyo, Japan. We analyzed the associations between reported parental time of returning home and the continuous Strengths and Difficulties Questionnaire (SDQ) scores using multivariable regression modeling.

**Results:** Children whose parents both returned home late (later than 6 p.m. for the mother and later than 8 p.m. for the father), or at irregular times, had higher scores in total difficulties (β = 1.20, 95% CI: 0.55 to 1.85), the “conduct problems” subscale (β = 0.37, 95% CI: 0.13 to 0.60), and the hyperactivity/inattention subscale (β = 0.53, 95% CI: 0.24 to 0.82) compared with children whose parents both returned home earlier. Mediation analyses indicated that the percentage of the total association between parental time of returning home and the SDQ scores, which was mediated by parent-child interaction, was 20% (95% CI: 10 to 46) for total difficulties, 17% (95% CI: 7 to 49) for conduct problems, and 23% (95% CI: 11 to 52) for hyperactivity/inattention.

**Conclusions:** Late or irregular returning home times for both parents had an adverse effect on child mental health, and the relationship was partly mediated by reduced frequency of parent-child interaction.

## Introduction

Globally, mental disorders are responsible for 8.5% of disability-adjusted life years among children aged 5–9 years old (1). Attention deficit hyperactivity disorders, oppositional defiant and conduct disorders, anxiety disorders, and depressive disorders are among the most common types of child mental disorders (2). In addition, these behavioral, emotional, and mental health issues in childhood later influence social and academic experiences (3, 4) with long-term consequences on adult health (4).

As the labor force participation rate for mothers with children increases in countries such as Australia, Europe, and North America, more researchers have explored the relationship between parental employment and child mental health (5). Empirical evidence indicates that only maternal full-time employment early in the life course is associated with more behavioral problems in children (5, 6), whereas maternal employment as a whole, which includes full-time and part-time employment, does not have strong effects on child mental health, and is deleterious only in socioeconomically advantaged communities (5), suggesting the protective effects of financial security provided by employment in households that are less stable socioeconomically.

Among dual-earner families, maternal and/or paternal working hours in the evening, night, or at irregular times have been shown to increase the risk of behavior problems in children (7, 8). Such parental working hours can lead to reduced quantity of parent-child interaction (9, 10), and poorer quality of parenting and home environments (9, 11), which are shown to have an independent impact on child mental health. Frequency of parent-child interaction was inversely related to child behavior problems (7). Parents and children sharing a variety of activities together has been suggested to reduce risk-taking behaviors among adolescents (9). Low-quality parenting practices (i.e., frequent unfavorable reactions to children's problem behaviors) have been associated with child difficulties (8, 12).

While most literature has assessed the influence of parental work schedules on child mental health, little research has directly examined the role of the time that parents return home from work. Several factors other than work schedules may affect the time that parents spend with their children. For example, 77% of workers in Japan had an average commute time of 79 min per day traveling to and from work, with the longest commuting time reported in the Tokyo area (13). In addition, 6% of workers in Japan participated in social activities with colleagues and business partners after work for an average of 118 min per week in 2015, and the proportion differed depending on sex and socioeconomic status (13). Parental time of returning home from work is possibly a more reasonable proxy of family environment influencing child mental health than parental time at work.

Just like other OECD countries, the labor force participation rate for mothers with children is increasing in Japan. In 2016, 54.0% of Japanese women with preschool children were engaged in work, an increase of 17.6% from 15 years earlier (14). Time spent working on a weekday increased slightly for employed Japanese women aged 30–39 years with children, rising from 376 to 393 min on average between 2001 and 2011 (15), respectively. However, the impact of parental working hours on child mental health in Japan has not been carefully studied.

In this study, we examined the association between parental time of returning home from work and child mental health among primary school students in Japan. We then estimated to what extent parent-child interaction mediates this association.

## Materials and methods

### A-CHILD data

We used data from the first wave of the Adachi Child Health Impact of Living Difficulty (A-CHILD) study, conducted by Adachi City Tokyo Medical and Dental University, and National Center for Child Health and Development, in Tokyo, Japan. Adachi City is located north of the center of Tokyo Metropolis. The overall rate of children receiving financial assistance for school supplies and school lunches for financial reasons in Adachi City was 35.8% (2015) and higher than the metropolitan average (21.6% in 2014), and the life expectancy in Adachi City (78.5 years for male and 85.4 years for female) was shorter than the metropolitan average (79.8 years for male and 86.4 years for female) in 2010. The A-CHILD study is an ongoing longitudinal study of children in public kindergartens and public elementary and junior high schools in Adachi City, Tokyo, and their families. The A-CHILD study was designed to examine the health status and living conditions of children, to investigate the impact of family environment and lifestyle on child health, and to understand the mechanisms through which household socioeconomic conditions influence child health. The first wave of the survey included first-grade children from all 69 public elementary schools. Data collection took place in July 2015 in six schools (pilot survey) and November 2015 in the other 63 schools (main survey). Data were collected using a parent/caregivers' questionnaire. The questionnaires were distributed to the children in class by the teachers, and the children took the questionnaire home for their parent/caregivers to fill out. The children then brought the completed questionnaire to school to submit to the teachers. Informed consent was obtained from the parent/caregiver by including a question at the beginning of the questionnaire about their participation agreement in the study. As of April 2015, there were 5,383 first-grade children in public elementary schools, of whom 28 either moved out of the city or were absent for long time at the time of the survey. Another 888 children did not submit the completed questionnaire, and the parents of 173 children refused to participate. Additionally, three children did not have any valid answers. Therefore, 4,291 children were included in the dataset.

The A-CHILD study was reviewed and approved by the Institutional Review Board at the National Center for Child Health and Development. This study was also approved by the Institutional Review Board at Tokyo Medical and Dental University.

### Outcome measure

The primary outcome for this study was parental report of child mental health problems, measured by the Japanese language version of the Strengths and Difficulties Questionnaire (SDQ), without impact supplement version, for the parents of 4–17 year olds available at http://www.sdqinfo.com. The SDQ consists of 25 child behavioral attributes that were divided into five subscales, namely emotional symptoms, conduct problems, hyperactivity/inattention, peer relationship problems, and prosocial behavior. Based on parents' ratings, five subscale scores and a score for total difficulties were calculated. To calculate the total difficulties score, all but the prosocial behavior subscale score was summed. Each subscale score can range from 0 to 10, and the total difficulties score can range from 0 to 40. Higher scores for the total difficulties score, emotional symptoms, conduct problems, hyperactivity/inattention, and peer relationship problems indicated a higher likelihood of psychological difficulties, whereas higher scores for prosocial behavior indicated the presence of prosocial behavior. The SDQ scores are strongly correlated with the Rutter scores and the Child Behavior Checklist scores (16, 17). Reliability and validity of the SDQ among Japanese school children has been previously documented (18, 19).

### Parental time of returning home

The time parents returned home from work was assessed by two questions for each parent: “What is your present employment status: full-time employment, part-time employment, self-employment, side work, other, or unemployment?” and, for parents who were employed, “What time do you usually return home from work: before 6 p.m., between 6 and 8 p.m., between 8 and 10 p.m., 10 p.m. or later, irregular time due to shift work, or irregular time due to frequent overtime?”. We first dichotomized time of returning from work into “late or irregular” (6 p.m. or later for mothers and 8 p.m. or later for fathers, or irregular times) and “not late” (before 6 p.m. for mothers and before 8 p.m. for fathers, or if the parent was unemployed). We used 6 p.m. for mothers and 8 p.m. for fathers as the cut-off point, because they were the national average returning home times for working females and males, respectively (13). Combinations of each returning home time was then classified as “both parents not late,” “father late or irregular,” mother late or irregular,” or “both parents late or irregular.”

### Parent-child interaction

We created a parent-child interaction score by combining the frequency of nine types of activities performed together between a child and parents (parental tutoring; playing sports; playing computer games; playing cards; talking about school; talking about socio-political issues; talking about recent TV programs; preparing meals; and going out) (0 = seldom; 1 = once or twice per month; 2 = once or twice per week; 3 = 3 or 4 times per week; 4 = almost every day) and frequency of the child eating dinner alone (3 = never; 2 = rarely; 1 = sometimes; 0 = always) (Cronbach's alpha = 0.61). The parent-child interaction score ranged from 0 to 39.

### Additional covariates

Child sex, child living together with siblings and grandparents, parental age, education, and employment, household income, respondent of questionnaire, and respondent's psychological distress [assessed using Kessler 6 (20)] were used as potential confounders in the analysis.

### Statistical analysis

All children living with both parents (*n* = 3,511) contained in the main survey data of the A-CHILD study were eligible for inclusion in the analyses. A child was excluded if data documenting any of the SDQ scores, the parental time of returning home, and/or any components of the parent-child interaction score were missing (*n* = 524). After excluding those with missing values, 2,987 children were included in the analyses.

First, we compared the characteristics among children with different parental time of returning home pattern by using chi-squared test. Second, we calculated the mean SDQ scores and parent-child interaction scores across the categories of parental time of returning home pattern, and then compared them by ANOVA. The effect sizes, i.e., the proportion of variability explained (ω^2^), for parental time of returning home pattern were estimated.

Third, a multiple linear regression model was used to examine the relationship between SDQ scores and parental time of returning home pattern. To explore the potential mediation of the associations by parent-child interaction, we added parent-child interaction scores to the regression models, and compared the regression coefficients from models with and without the parent-child interaction variable. Regression coefficients were adjusted for all the covariates. All missing covariates were given dummy coding. To determine the extent to which the association between SDQ scores and parental returning home times was mediated by parent-child interaction scores, we performed mediation analysis (separate analysis for each category of parental returning home times) (21). We first estimated controlled direct effect, natural indirect effect, and total effect using PARAMED command in Stata version 14 (StataCorp, 2015). Interaction effect between parental returning home times and parent-child interaction score were not considered in the analysis. The effect sizes, ω^2^, for parental time of returning home pattern were estimated. The proportion mediated by the parent-child interaction score was then estimated using the MEDIATE command in R version 3.4.0 (R Foundation, 2017). Nonparametric bootstrapping was applied to calculate *p*-values and 95% confidence intervals for the proportion that was mediated.

Further, we conducted sensitivity analyses of SDQ variables by categorizing the SDQ scores as normal, borderline, and clinical based on the distribution of the scores among Japanese children aged 4–12 years of age (22), and used multinomial regression models to assess the relationship between parental time of returning home pattern and the categorical SDQ scores.

## Results

Table 1 shows the demographic and socioeconomic characteristics summarized by the parental time of returning home pattern. Both mothers and fathers were not late returning home in 805 (27.0%) families, mothers were not late returning home and fathers returned late or at irregular times in 1650 (55.2%) families, mothers returned late or at irregular times and fathers were not late returning home in 171 (5.7%) families, and both mothers and fathers returned home late or at irregular times in 361 (12.1%) families. Mothers who returned home late or at irregular times (*n* = 532) were older (*p* = 0.004) and more educated (*p* = 0.014); and fathers who returned home late or at irregular times (*n* = 1,821) were younger (*p* ≤ 0.001) and more educated (*p* < 0.001). Annual income was higher (*p* < 0.001) and grandparents were more likely to live together with the family (*p* = 0.093) in households where mothers and/or fathers returned home late or at irregular times. Level of psychological distress was similar between parental time of returning home categories (*p* = 0.89).

**Table 1:** Descriptive statistics for the study sample by parental time of returning home pattern^*^.

	**All children(*n* = 2987)**	**Parental time of returning home pattern**					**Maternal time of returning home pattern**			**Paternal time of returning home pattern**		
		**Mother and father not late (*n* = 805)**	**Mother not late and father late or irregular (*n* = 1650)**	**Mother late or irregular and father not late (*n* = 171)**	**Mother and father late or irregular (*n* = 361)**	***p***	**Not late (*n* = 2455)**	**Late or irregular (*n* = 532)**	***p***	**Not late (*n* = 976)**	**Late or irregular (*n* = 2011)**	***p***
	**(%)**	**(%)**	**(%)**	**(%)**	**(%)**		**(%)**	**(%)**		**(%)**	**(%)**
**CHILD CHARACTERISTICS**												
Sex						0.379			0.088			0.918
Male	51.7	51.1	50.9	53.8	55.7		51.0	55.1		51.5	51.8	
Female	48.2	48.8	49.0	46.2	44.3		49.0	44.9		48.4	48.2	
Missing	0.1	0.1	0.1	0.0	0.0		0.1	0.0		0.1	0.0	
**MATERNAL CHARACTERISTICS**												
Age						< 0.001			0.004			0.002
< 35 years	21.5	25.1	21.0	19.3	16.6		22.3	17.5		24.1	20.2	
35–39 years	35.0	30.9	37.2	36.8	33.2		35.1	34.4		32.0	36.4	
40–44 years	32.3	32.0	32.1	25.1	37.1		32.1	33.3		30.8	33.0	
≥45 years	10.0	10.8	8.5	17.5	11.6		9.2	13.5		12.0	9.1	
Missing	1.3	1.1	1.3	1.2	1.4		1.3	1.3		1.1	1.3	
Education						< 0.001			0.014			< 0.001
Junior high school or high school	33.3	40.4	31.2	32.2	27.7		34.2	29.1		38.9	30.5	
Technical/junior college or college dropout	43.5	40.2	45.3	38.6	44.6		43.7	42.7		40.0	45.2	
Collage or graduate school	21.8	17.9	22.1	27.5	26.3		20.7	26.7		19.6	22.9	
Others	0.6	0.6	0.6	0.6	0.6		0.6	0.6		0.6	0.6	
Missing	0.8	0.9	0.8	1.2	0.8		0.8	0.9		0.9	0.8	
Employment status						< 0.001			< 0.001			0.055
Employed	61.9	56.8	52.1	100.0	100.0		53.6	100.0		64.3	60.7	
Not employed	38.1	43.2	47.9	0.0	0.0		46.4	0.0		35.7	39.3	
**PATERNAL CHARACTERISTICS**												
Age						0.003			0.220			< 0.001
< 35 years	13.6	14.0	14.1	11.1	11.9		14.1	11.7		13.5	13.7	
35–39 years	30.0	26.2	32.4	28.1	28.3		30.4	28.2		26.5	31.7	
40–44 years	33.8	33.4	33.6	31.6	36.8		33.5	35.2		33.1	34.2	
≥45 years	21.3	25.1	18.7	27.5	21.6		20.8	23.5		25.5	19.2	
Missing	1.3	1.2	1.3	1.8	1.4		1.3	1.5		1.3	1.3	
Education						< 0.001			0.301			< 0.001
Junior high school or high school	36.6	48.9	31.6	35.7	32.7		37.3	33.6		46.6	31.8	
Technical/junior college or college dropout	21.8	19.1	22.2	27.5	23.0		21.2	24.4		20.6	22.4	
Collage or graduate school	40.4	31.1	45.0	33.9	43.2		40.4	40.2		31.6	44.7	
Others	0.4	0.2	0.5	1.2	0.0		0.4	0.4		0.4	0.4	
Missing	0.8	0.6	0.7	1.8	1.1		0.7	1.3		0.8	0.7	
Employment status						< 0.001			0.570			<!0.001
Employed	99.0	96.9	100.0	97.7	100.0		99.0	99.2		97.0	100.0	
Not employed	1.0	3.1	0.0	2.3	0.0		1.0	0.8		3.0	0.0	
**HOUSEHOLD CHARACTERISTICS**												
Household income						< 0.001			< 0.001			< 0.001
<3,000,000 JPY	6.4	9.1	5.6	5.8	3.9		21.8	41.5		21.5	27.1	
3,000,000 JPY – 4,999,999 JPY	26.0	33.9	24.8	16.4	18.3		6.8	4.5		8.5	5.3	
5,000,000 JPY – 7,499,999 JPY	34.2	29.6	38.7	32.7	24.9		27.8	17.7		30.8	23.6	
≥7,500,000 JPY	25.3	18.4	23.4	36.3	44.0		35.7	27.4		30.1	36.2	
Missing	8.2	9.1	7.5	8.8	8.9		8.0	8.8		9.0	7.8	
Child living together with sibling						0.355			0.993			0.087
Yes	81.8	79.8	82.7	81.3	82.0		81.8	81.8		80.0	82.6	
No	18.2	20.2	17.3	18.7	18.0		18.2	18.2		20.0	17.4	
Child living together with grandparent						0.023			0.047			0.048
Yes	8.9	10.3	7.5	10.5	11.4		8.4	11.1		10.3	8.2	
No	91.1	89.7	92.5	89.5	88.6		91.6	88.9		89.7	91.8	
**RESPONDENT CHARACTERISTICS**												
Relationship with child						< 0.001			0.464			< 0.001
Mother	91.1	88.2	92.6	86.0	92.8		91.2	90.6		87.8	92.6	
Others	7.7	10.6	6.0	12.3	6.6		7.5	8.5		10.9	6.1	
Missing	1.3	1.2	1.4	1.8	0.6		1.3	0.9		1.3	1.2	
K6 psychological distress scale score						0.983			0.976			0.887
<5	72.7	72.2	72.9	73.7	72.3		72.7	72.7		72.4	72.8	
≥5	27.1	27.5	27.0	26.3	27.4		27.1	27.1		27.3	27.1	
Missing	0.2	0.4	0.1	0.0	0.3		0.2	0.2		0.3	0.1	

*The p-values were generated by chi-square tests. The missing category was excluded from each analysis*.

* *Time of returning home was categorized as “late or irregular” if it was 6 p.m. or later for mothers, 8 p.m. or later for fathers, or irregular and “not late” if it was before 6 p.m. for mothers, before 8 p.m. for fathers, or if the parent was unemployed*.

Table 2 presents mean SDQ scores and parent-child interaction score according to parental times of returning home. Using the cut-offs among Japanese children aged 4–12 years of age (19), 13.7% of children were in the clinical range for the total difficulties score, 9.8% for emotional symptoms, 13.4% for conduct problems, 12.5% for hyperactivity/inattention, 7.8% for peer relationship problems, and 13.7% for prosocial behavior. Children in households where both mothers and fathers returned home late or at irregular times showed higher score on conduct problems and hyperactivity/inattention than other groups (*p* = 0.024 and 0.008, respectively).

**Table 2:** Child Strengths and Difficulties Questionnaire score and parent-child interaction score by parental time of returning home pattern^*^.

	**All children (*n* = 2987)**	**Mother and father not late (*n* = 805)**	**Mother not late and father late or irregular (*n* = 1650)**	**Mother late or irregular and father not late (*n* = 171)**	**Mother and father late or irregular (*n* = 361)**	***P*-value by ANOVA**	**ω^2^**
	**Mean (SD)**	**Mean (SD)**	**Mean (SD)**	**Mean (SD)**	**Mean (SD)**		
Total difficulties	9.72 (5.23)	9.83 (5.04)	9.45 (5.26)	9.81 (5.09)	10.61 (5.46)	0.059	0.004
Emotional symptoms	1.91 (1.81)	1.94 (1.78)	1.89 (1.84)	1.80 (1.65)	1.98 (1.79)	0.76	0.000
Conduct problems	2.43 (1.83)	2.40 (1.75)	2.35 (1.82)	2.54 (1.93)	2.77 (1.94)	0.024	0.005
Hyperactivity/inattention	3.55 (2.31)	3.54 (2.29)	3.45 (2.28)	3.73 (2.28)	3.94 (2.47)	0.008	0.004
Peer relationship problems	1.83 (1.69)	1.95 (1.68)	1.76 (1.69)	1.75 (1.53)	1.92 (1.78)	0.19	0.002
Prosocial behavior	6.60 (2.03)	6.69 (1.97)	6.59 (2.06)	6.60 (1.94)	6.39 (2.00)	0.18	0.001
Parent-child interaction	21.44 (4.59)	21.89 (4.37)	21.53 (4.56)	20.65 (4.63)	20.38 (4.97)	< 0.001	0.010

*ω^2^: The point estimate of the effect sizes of parental time of returning home pattern on Child Strengths and Difficulties Questionnaire score and parent-child interaction score*.

* *Time of returning home was categorized as “late or irregular” if it was 6 p.m. or later for mothers, 8 p.m. or later for fathers, or irregular and “not late” if it was before 6 p.m. for mothers, before 8 pm for fathers, or if the parent was unemployed*.

Multivariable regression analyses (Table 3) indicated that parental time of returning home was associated with total difficulties, conduct problems, and hyperactivity/inattention after adjusting for covariates. Compared with children in households where both mothers and fathers did not return home late, children in households where both mothers and fathers returned home late or at irregular times had higher scores for total difficulties (β = 1.20, 95% CI: 0.55 to 1.85), conduct problems (β = 0.37, 95% CI: 0.13 to 0.60), and hyperactivity/inattention (β = 0.53, 95% CI: 0.24 to 0.82). However, such effects were not observed when only one parent returned home late or at irregular times. Table 3 also shows that the parent-child interaction score was significantly associated with the parental time of returning home pattern (β = −0.52, 95% CI: −0.91 to −0.13 for mothers not returning late and fathers returning late or at irregular times; β = −0.94, 95% CI: −1.71 to −0.17 for mothers returning late or at irregular times and fathers not late, and = −1.34, 95% CI: −1.94 to −0.75 for mothers and fathers returning late or at irregular times). After additional adjustment for the parent-child interaction score, the magnitude of the impact of the parental time of returning home pattern on the SDQ score decreased; for example, β = 0.95 (95% CI: 0.31 to 1.60) for total difficulties, β = 0.31 (95% CI: 0.07 to 0.54) for conduct problems, and β = 0.41 (95% CI: 0.12 to 0.69) for hyperactivity/inattention, although all of these three associations were still statistically significant. The effect sizes (ω^2^) of parental time of returning home pattern were 0.005 (95% CI: 0.000 to 0.010) on total difficulties, 0.003 (95% CI: 0.000 to 0.008) on conduct problems, and 0.003 (95% CI: 0.000 to 0.009) on hyperactivity/inattention before controlling for parent-child interaction score.

**Table 3:** Multivariable linear regression models for Child Strengths and Difficulties Questionnaire scores and a parent-child interaction score.

	**Model without parent-child interaction score**	**Model with parent-child interaction score**
	**β (95% CI)**	**β (95% CI)**
**OUTCOME: TOTAL DIFFICULTIES**		
Parental time of returning home pattern^†^	ω^2^ = 0.005	ω^2^ = 0.003
Mother not late and father late or irregular	−0.01 (−0.44, 0.42)	−0.11 (−0.53, 0.32)
Mother late or irregular and father not late	0.29 (−0.56, 1.13)	0.12 (−0.72, 0.95)
Mother and father late or irregular	1.20^***^ (0.55, 1.85)	0.95^**^ (0.31, 1.60)
Parent-child interaction score		−0.18^***^ (−0.22, −0.14)
**OUTCOME: EMOTIONAL SYMPTOMS**		
Parental time of returning home pattern^†^	ω^2^ < 0.001	ω^2^ < 0.001
Mother not late and father late or irregular	0.00 (−0.16, 0.15)	−0.01 (−0.16, 0.14)
Mother late or irregular and father not late	0.00 (−0.30, 0.30)	−0.02 (−0.32, 0.29)
Mother and father late or irregular	0.20 (−0.04, 0.43)	0.17 (−0.06, 0.41)
Parent-child interaction score		−0.02^*^ (−0.03, 0.00)
**OUTCOME: CONDUCT PROBLEMS**		
Parental time of returning home pattern^†^	ω^2^ = 0.003	ω^2^ = 0.002
Mother not late and father late or irregular	−0.01 (−0.17, 0.15)	−0.03 (−0.19, 0.12)
Mother late or irregular and father not late	0.14 (−0.17, 0.44)	0.09 (−0.21, 0.40)
Mother and father late or irregular	0.37^**^ (0.13, 0.60)	0.31^*^ (0.07, 0.54)
Parent-child interaction score		−0.05^***^ (−0.06, −0.03)
**OUTCOME: HYPERACTIVITY/INATTENTION**		
Parental time of returning home pattern^†^	ω^2^ = 0.003	ω^2^ = 0.002
Mother not late and father late or irregular	0.07 (−0.12, 0.26)	0.03 (−0.16, 0.21)
Mother late or irregular and father not late	0.26 (−0.12, 0.63)	0.17 (−0.19, 0.54)
Mother and father late or irregular	0.53^***^ (0.24, 0.82)	0.41^**^ (0.12, 0.69)
Parent-child interaction score		−0.09^***^ (−0.11, −0.07)
**OUTCOME: PEER RELATIONSHIP PROBLEMS**		
Parental time of returning home pattern^†^	ω^2^ < 0.001	ω^2^ < 0.001
Mother not late and father late or irregular	−0.07 (−0.21, 0.07)	−0.09 (−0.23, 0.05)
Mother late or irregular and father not late	−0.11 (−0.39, 0.17)	−0.14 (−0.42, 0.14)
Mother and father late or irregular	0.11 (−0.11, 0.32)	0.07 (−0.15, 0.28)
Parent-child interaction score		−0.03^***^ (−0.04, −0.02)
**OUTCOME: PROSOCIAL BEHAVIOR**		
Parental time of returning home pattern^†^	ω^2^ < 0.001	ω^2^ < 0.001
Mother not late and father late or irregular	−0.17 (−0.34, 0.00)	−0.11 (−0.28, 0.05)
Mother late or irregular and father not late	−0.01 (−0.35, 0.33)	0.09 (−0.24, 0.42)
Mother and father late or irregular	−0.20 (−0.46, 0.06)	−0.06 (−0.31, 0.20)
Parent-child interaction score		0.11^***^ (0.09, 0.12)
**OUTCOME: PARENT-CHILD INTERACTION**		
Parental time of returning home pattern^†^	ω^2^ = 0.006	
Mother not late and father late or irregular	−0.52^**^ (−0.91, −0.13)	
Mother late or irregular and father not late	−0.94^*^ (−1.71, −0.17)	
Mother and father late or irregular	−1.34^***^ (−1.94, −0.75)	

*** p < 0.001;

** p < 0.01;

* *p < 0.05*.

*Regression models include parental time of returning home pattern, parent-child interaction score, child sex, child living together with sibling and grandparent, parental age, education, and employment, household income, respondent of questionnaire, and respondent's psychological distress*.

*The effect sizes of parental time of returning home pattern, ω^2^, and their 95% confidence intervals were shown. ns: not significant and the lower limit cannot be calculated*.

† *Time of returning home was categorized as “late or irregular” if it was 6 p.m. or later for mothers, 8 p.m. or later for fathers, or irregular and “not late” if it was before 6 p.m. for mothers, before 8 p.m. for fathers, or if the parent was unemployed. In the analyses, “mothers and fathers did not return home late” was used as the reference group*.

Results of the mediation analyses (Table 4) suggested that the parent-child interaction score partially but statistically significantly mediated the association between the parental time of returning home pattern (both parents returning late or at irregular times vs. both parents not returning late) and child SDQ scores of total difficulties (proportion mediated = 20%, 95% CI: 10 to 46%), conduct problems (proportion mediated = 17%, 95% CI: 7 to 49%), and hyperactivity/inattention (proportion mediated = 23%, 95% CI: 11 to 52%).

**Table 4:** Mediation by parent-child interaction of the relationship between mother and father who returned home late or at irregular times vs. mother and father who did not return home late and child total difficulties, conduct problems, or hyperactivity/inattention score.

**Child mental health**	**Mother and father returned home late or at irregular times (reference: mother and father did not return home late)**^†^			**Proportion mediated (95% CI)**
	**Controlled direct effect**	**Natural indirect effect**	**Total effect**	
	**β (95% CI)**	**β (95% CI)**	**β (95% CI)**	
Total difficulties	0.95^**^ (0.31, 1.60)	0.24^***^ (0.12, 0.36)	1.20^***^ (0.55, 1.85)	0.20^***^ (0.10, 0.46)
Conduct problems	0.31^*^ (0.07, 0.54)	0.06^***^ (0.03, 0.09)	0.37^**^ (0.13, 0.60)	0.17^***^ (0.07, 0.49)
Hyperactivity/inattention	0.41^**^ (0.12, 0.69)	0.12^***^ (0.06, 0.18)	0.53^***^ (0.24, 0.82)	0.23^***^ (0.11, 0.52)

*** p < 0.001;

** p < 0.01;

* *p < 0.05*.

*Regression models include parental time of returning home pattern, parent-child interaction score, child sex, child living together with sibling and grandparent, parental age, education, and employment, household income, respondent of questionnaire, and respondent's psychological distress. Analyses were limited to the associations where the total effect was significant*.

† *Time of returning home was categorized as ‘late or irregular’ if it was 6 p.m. or later for mothers, 8 p.m. or later for fathers, or irregular and ‘not late’ if it was before 6 p.m. for mothers, before 8 p.m. for fathers or if the parent was unemployed*.

Sensitivity analysis, that is, multinomial regression analyses of the categorical SDQ scores, suggested that late parental time of returning home was also significantly associated with the increased risk of the clinical categories of total difficulties and conduct problems (*p* = 0.001 and *p* = 0.019, respectively), and both the borderline and clinical categories of hyperactivity/inattention (*p* = 0.027 and *p* = 0.004, respectively), although we found significant reduced risk of the borderline category of emotional symptoms (*p* = 0.043) (Supplementary Table S1).

## Discussion

This study explores the relationship between parental time of returning home and child mental health among primary school students in Japan. It is the first to examine the impact of parental time of returning home from work using a population-based study in Tokyo, Japan. While previous research has assessed the influence of parental work schedules on child mental health, little research has directly examined the role of the time parents return home from work.

Our results suggest that children in households where both mothers and fathers returned late or at irregular times have a higher risk of showing mental health problems, in particular, conduct and hyperactivity/inattention problems. These difficulties constitute externalizing problems, which can pose a substantial burden to individuals and their families (23–25). This effect was not apparent for emotional symptoms and relationship problems, which constitutes internalizing problems. In general, these different results between children externalizing and internalizing problems is consistent with other studies. Hsueh and Yoshikawa (26) found that working nonstandard schedules and variable shifts were associated with children externalizing but not internalizing problems. Vieira et al. (27) also found that maternal work-family conflict increased the risk of children externalizing but not internalizing problems, and the association was mediated by its adverse effects on the quality of the parent-child relationship. A study in the United States investigating the relationship between type of child care and child mental health suggests that longer hours spent at child care facilities and shorter hours at home was only adversely associated with externalizing problems (28). This study also demonstrates that the magnitude of the effects of parent-child interaction on externalizing problems (conduct problems and hyperactivity/inattention problems) was greater than the magnitude of the effects for internalizing problems (emotional symptoms and relationship problems). These results and previous studies would suggest that parental time of returning home from work and associated parent-child interaction would have stronger effects on externalizing than internalizing problems.

The results also showed that, to a small extent, parent-child interaction partially mediated the association between parental time of returning home, and conduct problems and hyperactivity/inattention problems. This finding is consistent with earlier studies that reported the mediation effect of quality and/or quantity of parent-child interaction on the relationship between parental working hours and child externalizing behavior problems (7–9). We are not able to determine the exact mechanisms of this association from our results, but some explanations might be possible. Evidence suggests that parental supervision is associated with lower risk of child externalizing behavior (9, 29–31). Parental monitoring efforts include frequent conversation with the child about the child's activities and friends (32), and our results suggest that frequency of such parent-child interaction was reduced in households where both parents returned home late or at irregular times. Second, absence of parents in the home and reduced frequency of interaction with parents might cause loneliness in children (33, 34), which induces externalizing behavior, whereby children seek attention from the parent (35).

As shown in the sensitivities analyses, parental returning home late or at irregular time might have stronger effects on the risk of clinically severe child difficulties than on borderline level. Thus, although the effect size was small in the linear analysis, we confirmed that later parental retuning home may have clinical impact on behavior problems of the offspring. We also found reduced risk of borderline level of emotional symptoms, which might be because parents retuning home late or at irregular time may not be able to detect borderline level of emotional symptoms of children. Further research is needed to confirm the association using longitudinal study.

It should be noted that the effect sizes of parental time of returning home pattern on child mental health was small, that is, later parental time of returning home had impact for 0.5% or less on child behavior problems. Previous observational studies on risk of SDQ among children also reported smaller effect size. For example, a previous study of the associations between early childhood fish and processed food consumption and conduct problems assessed by SDQ reported the effect sizes of η^2^ = 0.001, which can be interpret that effect of fish and processed food explained 0.1% of child SDQ (36). Thus, although the impact of late or irregular parental time retuning home is small, it cannot be ignored as other important risk factors on child behavior problems showed similar effect size.

This study has a few limitations. First, we used parental reports of returning home times, which may contain measurement errors due to self-reporting. Second, this study does not address the use of child care services after school. Additional adjustment for use of child care services, therefore, might the magnitude of the adverse effect of parental time of returning home reported in this study. Third, this study uses cross-sectional data that cannot account for changes in parental time of returning home and child mental health over time, meaning that reverse causation is likely. Some evidence also suggests that parental employment can affect child mental health through parental well-being (6), especially parental depression, which is related to adverse working conditions. Our observation that the magnitude of the effects (i.e., the regression coefficient in multivariable linear models) for both parents being late or returning home at irregular timing category reduced by about 5 to 20% (10.8% for total difficulties, 15.8% for emotional symptoms, 8.6% for conduct problems, 7.6% for hyperactivity/inattention, 22.3% for peer relationship problems, and 5.0% for prosocial behavior) after adjusting for categorical variable of K6 psychological distress scale score (results not shown), supports this possible mechanism. However, we did not consider psychological distress of respondents as a mediator in the main analyses because psychological distress of respondents might influence their assessment of child mental health, as well as time of returning home for both parents. Therefore, this hypothesis is difficult to examine by cross-sectional analyses. To further understand this hypothesized mechanism, future research should include a questionnaire on parental work stress and investigate the impact of change in the parental time of returning home on change in parental and child mental health.

In conclusion, children whose parents both returned home late or at irregular times have a higher risk of showing conduct problems and hyperactivity/inattention problems, and the relationship was partially mediated by reduced parent-child interaction. Future research should explore the variety of positive and negative reasons why parents returned home late that relate to the household environment.

## Author contributions

TF conceived the study. MO, AI, TK, and TF conducted the survey. MK analyzed data and wrote first draft. TF finalized manuscript. All authors approved the final manuscript.

### Conflict of Interest Statement

The authors declare that the research was conducted in the absence of any commercial or financial relationships that could be construed as a potential conflict of interest.
