# Supplementary material for: Parental Time of Returning Home From Work and Child Mental Health Among First-Year Primary School Students in Japan: Result From A-CHILD Study
Source: Front Pediatr. 2018 Jul 2;6:179. doi: 10.3389/fped.2018.00179 (PMC6036177; doi:10.3389/fped.2018.00179)
Supplement: Supplementary file 1 [file Table_1.docx]

**Supplementary Table S1**

Multivariable multinomial regression models for categorical Child Strengths and Difficulties Questionnaire scores

| Parental time of returning home pattern† | Child Strengths and Difficulties Questionnaire category‡ (reference: normal) | | | | | |
| --- | --- | --- | --- | --- | --- | --- |
|  | Borderline | | | Clinical | | |
|  | OR | 95% CI | p | OR | 95% CI | p |
| Outcome: Total difficulties | | | | | | |
| Mother not late and father late or irregular | 0.97 | 0.75-1.26 | 0.83 | 1.07 | 0.81-1.41 | 0.63 |
| Mother late or irregular and father not late | 1.10 | 0.67-1.80 | 0.70 | 1.20 | 0.71-2.03 | 0.51 |
| Mother and father late or irregular | 0.76 | 0.50-1.17 | 0.22 | 1.87 | 1.28-2.73 | 0.001 |
| Outcome: Emotional symptoms subscale | | | | | | |
| Mother not late and father late or irregular | 0.83 | 0.61-1.12 | 0.22 | 1.19 | 0.87-1.61 | 0.27 |
| Mother late or irregular and father not late | 1.17 | 0.66-2.07 | 0.59 | 0.93 | 0.48-1.81 | 0.83 |
| Mother and father late or irregular | 0.58 | 0.34-0.98 | 0.043 | 1.34 | 0.85-2.13 | 0.21 |
| Outcome: Conduct problems subscale | | | | | | |
| Mother not late and father late or irregular | 1.18 | 0.88-1.57 | 0.27 | 1.06 | 0.81-1.40 | 0.65 |
| Mother late or irregular and father not late | 1.10 | 0.63-1.92 | 0.74 | 1.55 | 0.95-2.52 | 0.08 |
| Mother and father late or irregular | 1.38 | 0.91-2.10 | 0.13 | 1.58 | 1.08-2.33 | 0.019 |
| Outcome: Hyperactivity/inattention subscale | | | | | | |
| Mother not late and father late or irregular | 1.07 | 0.76-1.51 | 0.69 | 1.10 | 0.83-1.46 | 0.50 |
| Mother late or irregular and father not late | 1.52 | 0.84-2.75 | 0.17 | 1.22 | 0.71-2.09 | 0.47 |
| Mother and father late or irregular | 1.70 | 1.06-2.73 | 0.027 | 1.78 | 1.20-2.64 | 0.004 |
| Outcome: Peer relationship problems subscale | | | | | | |
| Mother not late and father late or irregular | 0.80 | 0.58-1.10 | 0.17 | 0.98 | 0.70-1.36 | 0.89 |
| Mother late or irregular and father not late | 0.37 | 0.15-0.88 | 0.025 | 0.84 | 0.42-1.64 | 0.60 |
| Mother and father late or irregular | 1.09 | 0.68-1.75 | 0.72 | 1.10 | 0.67-1.82 | 0.71 |
| Outcome: Prosocial behavior subscale | | | | | | |
| Mother not late and father late or irregular | 1.07 | 0.84-1.35 | 0.60 | 1.27 | 0.97-1.67 | 0.08 |
| Mother late or irregular and father not late | 0.84 | 0.52-1.34 | 0.46 | 0.81 | 0.47-1.41 | 0.46 |
| Mother and father late or irregular | 0.95 | 0.67-1.37 | 0.80 | 1.31 | 0.89-1.93 | 0.17 |

Odds ratios were adjusted for child sex, child living together with sibling and grandparent, parental age, education, and employment, household income, respondent of questionnaire, and respondent’s psychological distress.

†Time of returning home was categorized as ‘late or irregular’ if it was 6 pm or later for mothers, 8 pm or later for fathers, or irregular and ‘not late’ if it was before 6 pm for mothers, before 8 pm for fathers, or if the parent was unemployed. In the analyses, “mothers and fathers did not return home late” was used as the reference group.

‡Child Strengths and Difficulties Questionnaire scores were categorized as normal, borderline, and clinical (total difficulties: 0-12, 13-15, and 16-40; emotional symptoms: 0-3, 4, and 5-10; conduct problems: 0-3, 4, and 5-10; hyperactivity/inattention: 0-5, 6, and 7-10; peer relationship problems: 0-3, 4, and 5-10; and prosocial behavior: 6-10, 5, and 0-4, respectively).
